# Experimental Study on the Effects of Coolants on Surface Quality and Mechanical Properties of Micromilled Thin-Walled Elgiloy

**DOI:** 10.3390/ma11091497

**Published:** 2018-08-22

**Authors:** Da Qu, Peng Zhang, Jiadai Xue, Yun Fan, Zuhui Chen, Bo Wang

**Affiliations:** 1 Centre for Precision Engineering, Harbin Institute of Technology, 92 West Dazhi Street, Nan Gang District, Harbin 150001, China; bennyqu007@yahoo.com (D.Q.); brucexjd@hit.edu.cn (J.X.); 2 Manufacturing Department, 618 Flight Automatic Control Research Institute, 92 Dianziyi Road, Yanta District, Xi’an 710065, China; zzbfactory@facri.com (Y.F.); 15765517664@163.com (Z.C.)

**Keywords:** mechanical properties, tool wear, tensile test, micromilling, MQCL method, cooling effect

## Abstract

In this study, minimum quantity coolant/lubrication (MQCL) is found to have significant impact on the surface quality and mechanical properties of the micromilled thin-walled work piece that is the core component of an aeroaccelerometer. Three kinds of coolants were used in the micromilling process to analyze their effects on surface quality and mechanical properties of the component. The experiment results show that an appropriate dynamic viscosity of coolant helps to improve surface roughness. The high evaporation rate of the coolants can enhance the cooling performance. Comparing with the dry machining case, MQCL has better performance on improving tool wear, surface quality, and mechanical properties of the micromilled work piece. It yielded up to 1.4–10.4% lower surface roughness compared with the dry machining case in this experiment. The machined work piece with the best mechanical properties and the one with the worst mechanical properties appeared in the ethyl alcohol and the dry machining case, respectively. The reasons for deteriorating surface quality and mechanical properties in dry machining cases are also analyzed. For improving the micromilling process, the penetration and cooling effect of the coolants are more important. This paper gives references to obtain better service performance of the component by improving the micromilling process.

## 1. Introduction

Lubrications/coolants as necessary auxiliary means can effectively enhance tool life and surface quality in the machining process. They mainly include minimum-quantity coolant/lubrication (MQCL), high pressure air/coolant (HPA/HPC), flood cooling, nanoparticles, and cryogenic cooling [1]. For different kinds of materials and machining methods, various auxiliary means are analyzed in precision/ultraprecision machining in recently years [2]. In machining hard-to-machine material, cryogenic cooling is always used as an effective cooling means. A combination of CO_2_-snow and MQL have been used to improve chip breaking in turning titanium alloys. However, CO_2_ coolant is not recommended due to its greenhouse pollution [3]. Even though a cryogenic condition has the best improvement effect on tool wear and cutting force, the best surface roughness is generated under an MQL condition [4]. For machining heat-resistant titanium aluminides, cryogenic cooling is the most effective method to improve surface roughness, and MQL the second. It is also promising to decrease tool flank wear [5]. For micromilling soft polymer material under cryogenic conditions, high rotational speed and small machining depth decrease surface marks [6]. However, cryogenic cooling is not suitable for machining all hard-to-machine materials in improving tool wear and surface quality, because tool rake face wear increases seriously, such as in micromilling Inconel 718 [7].

Flood cooling and HPA are not appropriate to use in micromilling thin-walled components [8]. Flood cooling is not so obvious at higher cutting speeds because of the bubble barrier or seizure effect, which hinders thermal transfer. Besides, it is always used in micromilling [9] and has been proven inferior to MQCL [10] in micromilling, including cost, environmental awareness, and making diseases [11]. HPA is not suitable with lower cutting speeds because it results in higher values of surface roughness and tensile residual stresses. Although HPA can decrease the generated heat between tool and work piece or chip in machining process, the highest tensile residual stress [8] and higher surface hardness [12] can be generated. Thus, it cannot be used in a micromilling process, especially when using a tool with only a several-hundred-microns diameter due to the low stiffness of the tool that can decrease surface accuracy [13]. 

Among the above cooling methods, MQCL has been accepted as the most effective one considering its cost, pollution, and performance [14]. It has good performance on decreasing tool wear [1,8,15] and surface roughness [12,16]. With MQCL, vegetable or mineral oil is used as common lubrication and bare coolants are discussed in milling, especially on a microscale. In 2010, the effect of MQL was firstly systematically analyzed in near micromilling [17]. Increasing oil flow rate was found to be not obvious to improve tool wear but increasing air-flow rate, and the distance from nozzle to the cutting zone also effects the size and penetration of the droplets [18]. In addition, MQCL was found to be affected by machining parameters as well. Pervaiz et al. [19] have found that the coolant effect firstly decreases and then increases as the increase of cutting speed. For analyzing tool wear under MQCL conditions, Liao et al. have found that MQL can provide extra oxygen to form a stable oxidation layer, such as SiO_2_ and Al_2_O_3_, between the tool-chip interface, and then decrease tool wear in turning mold steel [11]. Chetan et al. [20] have analyzed tool wear in turning two kinds of aerospace alloys. They have found that a smaller contact angle between droplets and work-piece surface makes more complete protective films and has a better lubrication effect. In conclusion, MQCL improves the machining process comprehensively. Hence, this paper discusses the performance of different coolants by MQCL in micromilling thin-walled components to near 10 μm thickness.

**Figure 1:**
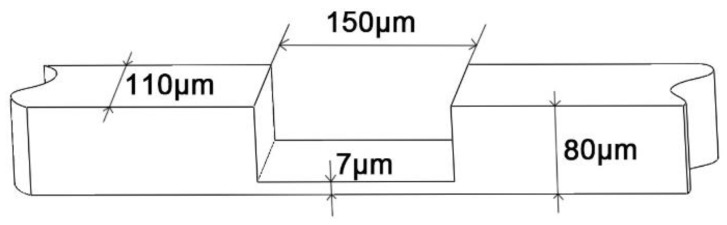
The thin-walled structure of the component.

With the lubrication/coolants method, most research takes only one-step micromilling with certain machining parameters in their experiments. As the work piece in this paper is very thick (see Figure 1), a multistep micromilling operation that contains similar machining craft was taken. The main difference is that each time the micromilling step generates corresponding residual stress on the surface and subsurface with which the next-step micromilling operation is also influenced. The final surface textures and mechanical properties of the work piece are the comprehensive consequence that is generated by the complex multistep machining in the process. Differing from the research mentioned above, three different kinds of MQCL coolants and dry micromilling operations are compared in this experiment. It reveals the influence of their different physical properties on surface quality and mechanical properties of the machined work piece, and is also an aspect to indirectly study surface/subsurface hardening and residual stress. In this paper, Isopar H, ethyl alcohol, and distilled water are chosen and the reason is illustrated in Section 2.3. The machining results are also compared with the dry machining case. The machined surface trait and tool wear are analyzed. These are shown in Section 3.1 and Section 3.2. In Section 3.3, the influence of coolants on mechanical properties is discussed. In addition, as mechanical properties of the work piece are the most important factors that affect the service performance of the component, how the physical factors and cooling process affect the mechanical properties is discussed.

## 2. Materials and Methods

### 2.1. Hardware System and Milling Tool for Micromilling

**Figure 2:**
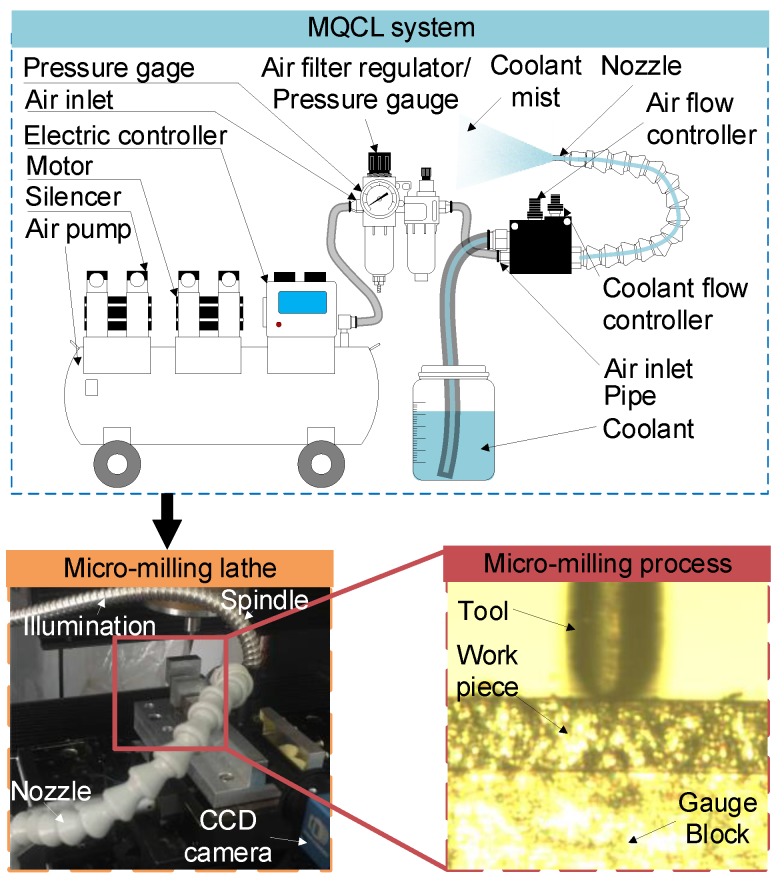
Micromilling machine tool.

A three-axis micromilling machine tool developed independently and an MQCL system, as shown in Figure 2, were used in this experiment. The employed spindle is manufactured by the British Loadpoint company. It is a high-speed air float electric spindle with less than 0.125 μm axial and radial run-out. The gratings for the three linear axes possess a resolution of 5 nm after subdivision. The precision of feed drives is 150 nm. A CCD camera (DAHENG, Beijing, China) was equipped onto the machine tool to set the milling tool. The milling tool was two-fluted end mills and its related information is listed in Table 1. 

### 2.2. Materials

For the core component of military accelerometer, the Elgiloy alloy is an ordinary material. It is a cobalt-based alloy with excellent physical performance, such as high strength, high ductility and low thermal conductivity, and chemical performance [21]. The Elgiloy used in this experiment is made in America and its original thickness is about 80 μm. The related composition and mechanical properties are shown in Table 2 and Table 3, respectively.

### 2.3. Experimental Method and Coolant Selection

When spindle rotational speed exceeds 40 krpm, the machined surface roughness under an MQCL condition is not smaller than that under a dry-machining condition. This is caused by insufficient lubrication [22]. The adsorption capacity of droplets sprayed from nozzle onto a rotating tool surface is determined by the relationship between surface tension and centrifugal force of lubricant [23]. A smaller diametrical tool generates a smaller centripetal force when the rest physical quantities stay constant. In this experiment, 40 krpm spindle rotational speed is used because the tool diameter is 150 μm, one quarter of 600 μm [23], with which the relative centripetal force reduces three times. Considering a micromilling thin-walled component, the multistep milling method [24] is used to remove a targeted removal depth 65 μm, which is separated into 10 μm of five times, 5 μm of two times, and 1 μm of five times. The complete experimental parameters are listed in Table 4.

Isopar H, ethyl alcohol, and distilled water were used in this experiment, and dry micromilling was operated as well. Isopar H is an effective lubricant due to its suitable dynamic viscosity and surface tension. In Reference [25], the water in OoW can suppress thermal expansion effort during the machining process. Hence, distilled water is also selected as a comparative coolant.

The amount of coolants is chosen as 15 mL/h, which accords with the requirement of the MQCL method [26]. They were separated into three stages ranging from level 1 to 3 with respect to milling length and were accepted as a three-times repeated experiment for next-step comparative analysis. To avoid the influence from tool wear and different coolants, the experiment is thus designed as seen in Table 5 due to each short-distance milling of using different coolants. A sharp tool was used for the cases of using coolants and should be flushed by the corresponding coolant in the interval of each experiment to avoid the influence from various coolants. The other one is only used for the dry-milling case and the related stages are selected as shown in Table 5 for the three levels.

### 2.4. Hardware System for Testing Mechanical Properties

**Figure 3:**
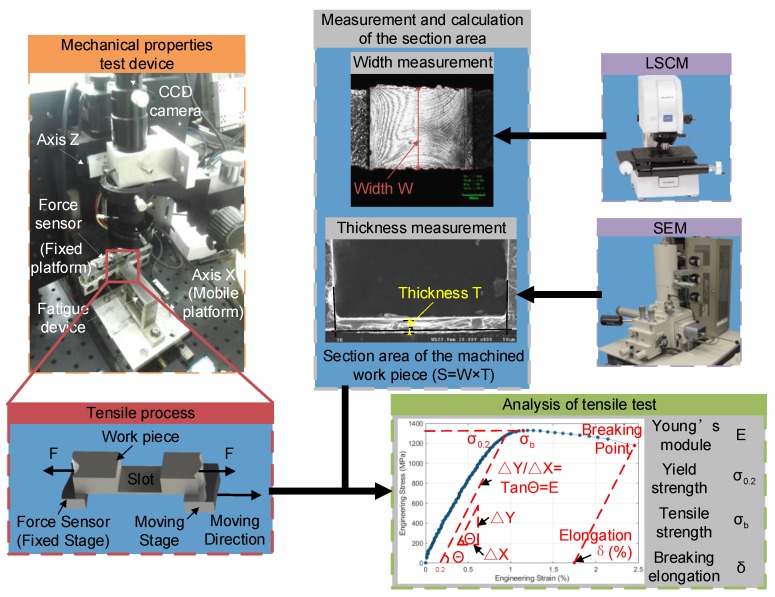
Sketch of testing mechanical properties.

A two-axis machine tool developed independently was used for testing the mechanical properties of thin-walled components. A force sensor, deemed as a fixed stage, is used for measuring tensile force and its force resolution is about 20 mN. The other stage is driven by motor to move at a very slow and constant speed by which the whole tensile test can be accepted as a quasistatic process. Orienting and holding the components can be operated by a CCD camera and adhesive, respectively. The adhesive should generate very little heat during its solidification. As the device can only obtain the relationship between displacement and force applied on one side of the tested component, the structure of the tested component should be measured in order to calculate the corresponding stress. The thickness of the machined component is calculated by analyzing pixels in an SEM image and the width of the component is obtained with the same method in an LSCM image. The machine tool and its testing process are shown in Figure 3.

## 3. Results and Discussion

### 3.1. Effect on Surface Quality

**Figure 4:**
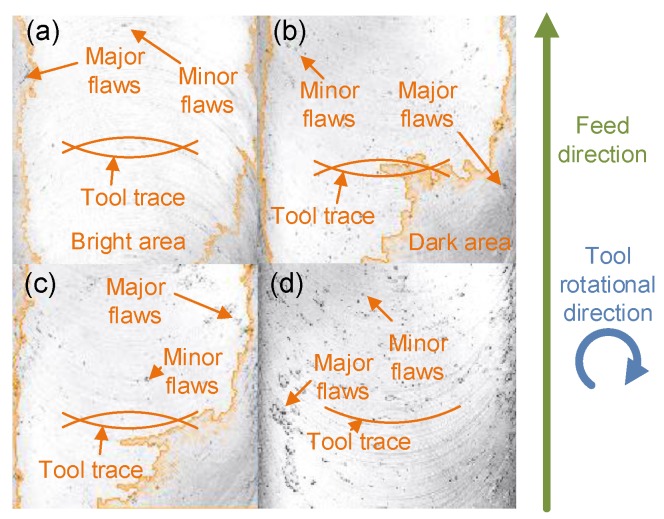
Surface textures of work piece micromilled under different coolants and methods. (**a**) Using Isopar H in MQCL; (**b**) using ethyl alcohol in MQCL; (**c**) using distilled water in MQCL; (**d**) under a dry-milling condition.

Surface integration mainly includes surface texture (mainly surface roughness), a metallurgical layer, and residual stress (RS) [8,27]. Figure 4 shows surface textures micromilled with the coolants mentioned above. Tool traces are imprinted on all the machined surfaces, especially on the areas that approach both sides of the slot base. As tool traces were generated by the adhesion of the work piece surface to the tool rake and flank faces causing the generation of built-up edge (BUE) and build-up lines [8], using MQCL cannot completely eliminate BUE, which is also a major factor that affects surface integrity. In Figure 4, the surface consists of a dark and bright area whose boundary is marked as orange. The surface morphology is captured by Laser Scanning Confocal Microscopy (LSCM), so the degree of brightness reflects the height of surface points and can also indirectly reflect surface roughness and surface accuracy. As a tool with a diameter of nearly 150 μm is employed, the stiffness in feed direction of the tool is very low and large deflection appears in the micromilling process [28], especially when using a large feed rate. Hence, the area that approaches both sides of the slot base is a little higher than other areas. In the case of using Isopar H, the machined surface shows with a minimum of flaws, which mainly form as adhered chips [29]. The dark area in the case of using ethyl alcohol is much larger than that in the case of using Isopar H, but approaches that in the case of using distilled water, which can be observed in Figure 4. One of the performances of MQCL is owing to the lubrication effect that changes the tribological properties and contact stress [30]. As the relationship between the dynamic viscosity of Isopar H ($V_{p}$ = 1.8 mm^2^/s), ethyl alcohol ($V_{e}$ = 1.4 mm^2^/s), and distilled water ($V_{d}$ = 0.9 mm^2^/s) is *V_p_* > *V_e_* > *V_d_* at 25 °C, large dynamic viscosity helps to decrease the friction across the area of the tool–chip interaction [8] and heat generation. Even though lower friction is good to reduce cutting force, penetration of coolants is more important [14]. However, much larger dynamic viscosity of coolants limits its penetration [31], even though the dynamic viscosity will decrease in the cutting zone due to high temperature [14]. Too higher dynamic viscosity decreases the flow of coolants and makes coolants activate the affinity to the cutting tool without extraction of the heat generated, and even has negative impact on cutting tool [7]. 

In general, coolant penetration decreases with increasing tool-chip contact length and higher surface tension [20]. In this experiment, Isopar H works to improve surface quality, and, thus, its dynamic viscosity is acceptable to penetrate. Compared with a surface machined with dry milling, the amount of minor flaws is much more than that of the previous three cases, especially the case of using Isopar H and ethyl alcohol. It shows that a larger dynamic viscosity of coolants also helps to decrease minor flaws on the surface during the machining process. In the case of dry milling, there are smeared chips, classified as major flaws, on the slot base surface, especially on the side surfaces of the slot base. In terms of the multistep micromilling method employed in the experiments, chips adhering on the tool in the previous step squeeze between the work piece and tool during the next-step milling process. Under these newly formed conditions, the adhering chips and the work piece are interacting due to BUE [29]. The loss of sharpness even increases the tool edge radius and cutting force during the cutting. Therefore, with the passing of the tool, the surface becomes deformed by rubbing due to the size effect [32], and surface stresses also occur. Surface integrity is then deteriorated by machining without using MQCL. Without liquid layer forms, a high amount of temperature and friction are generated [33]. Hence, coolants with scoped higher dynamic viscosity are recommended if better surface integrity is required. Among these coolants in this experiment, Isopar H and ethyl alcohol are the best choice for better surface integrity.

**Figure 5:**
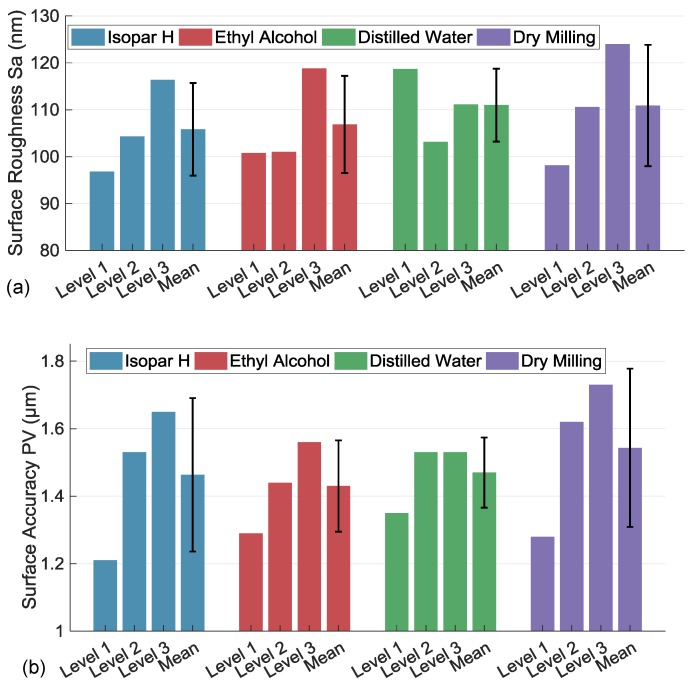
Surface quality of work piece micromilled under different coolants and methods. (**a**) surface roughness of the work piece; (**b**) surface accuracy of the work piece.

Figure 5a shows statistical surface roughness in the four cases. They are calculated from data captured by LSCM (OLS-3000) after filtering. On the same level, the three cases with coolants show lower surface roughness than that in the dry case. Dry micromilling accelerates tool wear as the cutting length increases. A BUE on tool forms more easily [23], and considering the material of the tool and work piece, Ni and Fe diffuse into WC, by which tool wear accelerates [11]. Hence, the machined surface with the best roughness is generated with Isopar H, and the surface roughness in the case of ethyl alcohol approaches that in the case of distilled water. 

As can be seen from Figure 5b, the statistical surface accuracy PV values also show that dry machining generates the worst surface accuracy. There is no cooling and lubrication effect to decrease heat between the interface of the tool rake face and chip, and in the interface of the tool flank face and machining surface, tool wear appears more dramatically. Besides that, the adhesive chip that is formed in previous machining step equivalently enlarges the tool edge radius and further generates the BUE on the surface to deteriorate surface roughness and accuracy. Unlike dry micromilling, surface accuracy generated with coolants is also decreased along with surface roughness. As the machining speed, which mainly affects surface roughness [34], is used in this experiment, surface roughness is then affected by coolants and MQL yields up to 1.4–10.4% lower value of surface roughness, compared with the dry-machining case. Referring to the lower value of 67% in reference [12], comparatively high feed per tooth also decreases the cooling and lubrication performance of MQCL. In addition, dry milling increases the hardness of the work piece surface, thus adversely affecting cutting forces, and consequently causing higher surface roughness in magnitude as well as in variations [35], especially using the multistep milling method. From the statistical PV values, the best surface accuracy appears in the case of using ethyl alcohol. 

### 3.2. Observation of Tool Edge

**Figure 6:**
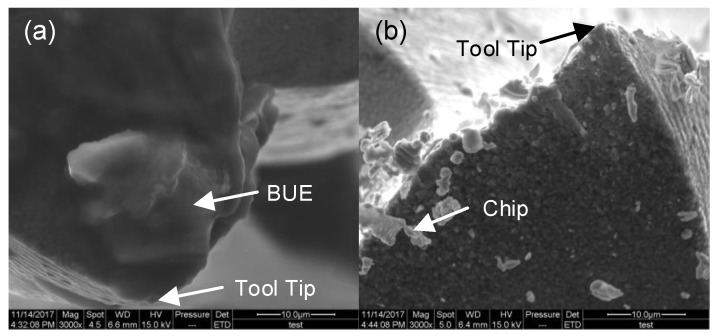
Scanning Electron Microscope (SEM) image of tool surfaces. (**a**) In the dry micromilling case; (**b**) in the MQCL micromilling case.

Figure 6 shows the tool-tip images captured by Scanning Electron Microscope (SEM) (Hitachi S-4300, Japan) and their length scales are both 10 μm. In Figure 6a, BUE was obviously found on the tool surface. But in Figure 6b, there were only some chips and no obvious BUE on the tool surface. As can be seen from the status of the chips on the tool surface, the chips do not strongly adhere to the tool surface and can drop out from the tool surface comparatively easily. This status will reduce the chance of forming smeared chips in the next machining step as well. The result also shows that MQCL can effectively reduce the chance of forming BUE by taking away heat generated in the primary deformation zone and tool-work piece interaction [8] during the machining process. Comparing Figure 6b with Figure 6a, the tool edge radius in the MQCL case is signally smaller than that in the dry-machining case even though the whole cutting length is not large. The cutting performance will decrease due to the BUE and thus increase tool wear [1]. Besides, the high ductility property of this work piece also accelerates tool wear [15]. In a word, MQCL has the auxiliary ability to prolong tool life. 

### 3.3. The Effect of the Coolants on the Mechanical Properties

The deformed layer and RS are the main factors that affect tensile strength and fatigue strength. Compressive residual stress (CRS) enhances fatigue life and tensile strength [27], and the RS can be measured by high-energy X-ray diffraction [31]. However, this measurement method cannot be used in a slot with only 150 μm width due to using a large facula [36]. The final service performance is decided by the mechanical properties. Thus, mechanical properties can reflect service performance more directly and reflect RS indirectly. As the influence of RS on tensile strength is obvious [27] and the mechanical properties are the key indexes, the mechanical properties of the machined work piece are analyzed directly. The main mechanical properties [37], which include Young’s module *E*, yield strength $\sigma_{0.2}$, tensile strength $\sigma_{b}$, and breaking elongation δ, are calculated from engineering the stress-engineering strain curve of the test component. An original component is tested for calibrating related calculation parameters. They are set as criteria for subsequent tests and the standard mechanical properties are listed in Table 6.

#### 3.3.1. The Effect of the Coolants on Young’s Module

**Figure 7:**
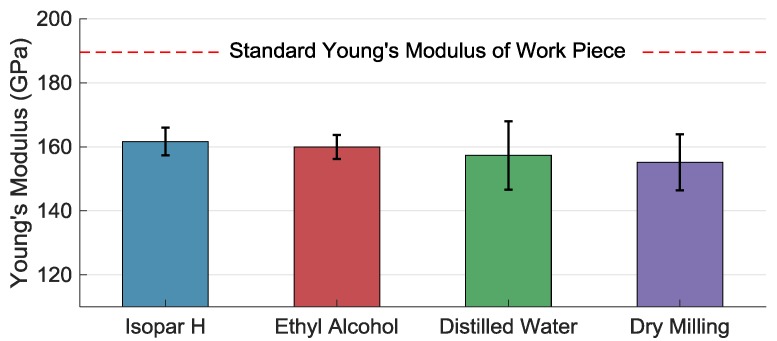
Young’s module of work piece micromilled under different coolants and methods.

Young’s module *E* of this component decides the sensitivity and measurement range of the accelerometer. In Figure 7, Young’s modules under different coolant methods are all smaller than the standard Young’s module. Dry milling always produces the highest microhardness [38] caused by strong strain hardening and deformed layer that impacts on the elastic module [39]. This is the combination of a high strain gradient and thermal gradient. Besides, the increase of surface microhardness [35] in the previous milling step enhances milling force and thermal concentrate in the current milling step while using the multistep milling method. It is a process of repeated influences. In reference [40], the microhardness (HV) and nanohardness (HN) of Al-Al_2_O_3_ nanocomposite increase with the increase of ball-milling time, and its Young’s module has positive correlation with its HV and HN. However, in this experiment, there is no obvious relationship between surface hardness and Young’s module. The thickness of the deformed layer is decreased by the multistep method but increased by comparatively high machining speed [31]. It is always at a microscale [8] and the whole thickness of the machined component is only near 15 μm, so the differences between Young’s module *E* under different cases are not small. 

#### 3.3.2. The Effect of the Coolants on Yield Strength

**Figure 8:**
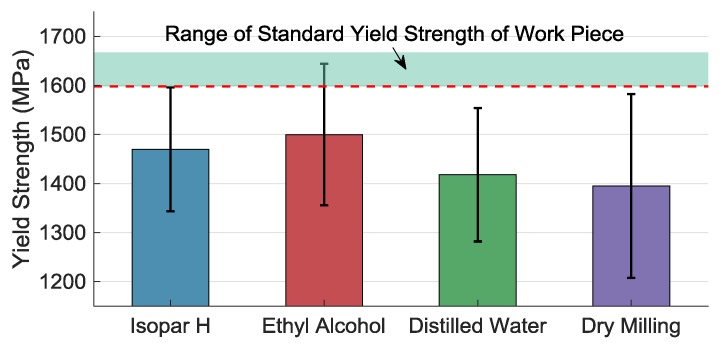
Yield stress of work piece micromilled under different coolants and methods.

Yield strength $\sigma_{0.2}$ of the component indirectly reflects the service performance of the accelerometer. Figure 8 shows that $\sigma_{0.2}$ of the all components are smaller than the standard one. The group that possesses the largest yield strength is machined with ethyl alcohol. As the temperature of the machined surface is higher than that of deeper layer in the micromilling process, the machined surface then stands tensile residual stress. When it is effectively decreased by using ethyl alcohol as coolant, the tensile residual stress of the machined surface is decreased as well and the yield strength is thus comparatively increased. However, it is still smaller than the standard yield strength because of the deformed layer. The machined thin-walled work piece with better surface quality possesses larger yield stress.

#### 3.3.3. The Effect of the Coolants on Tensile Strength

**Figure 9:**
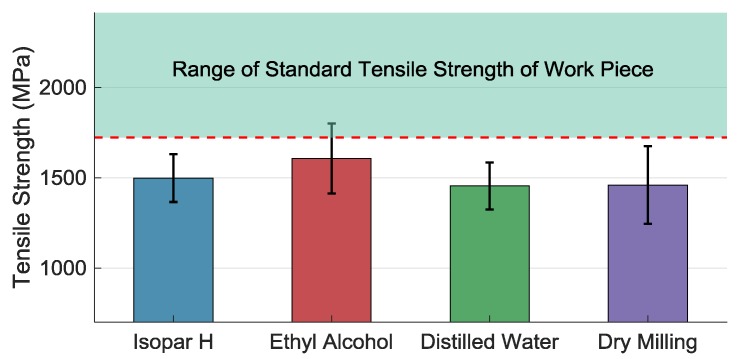
Tensile strength of work piece micromilled under different coolants and methods.

Tensile strength $\sigma_{b}$ of the machined work piece directly decides the service performance of accelerometer, so it is a very important index. Figure 9 shows the tensile strength under different coolant methods. The $\sigma_{b}$ of all the machined work piece is smaller than that of the original material. The largest tensile strength appears at the work piece micromilled with ethyl alcohol. Tensile strength is mainly affected by two factors: deformed layer and RS. CRS is generated by mechanical load [31], especially when micromilling with a large negative rake angle [41]. However, tensile residual stress (TRS) is generated by high temperature [42]. As TRS is mainly affected by heat in the milling process, the cooling effect of MQCL is the main factor by which TRS is reduced [43] compared with the dry-milling case. In the dry-milling case, tool wear accelerates and tool edge radius increases, by which rubbing and ploughing [44] happens more easily. It is an abusive machining condition leading to bad surface integrity [45]. Compressive stress and heat are generated very fast. This will also cause an increase in the RS under the surface [46]. Obviously, according to the experimental results, heat generation is more concentrated. This is another factor of MQCL works, which alters heat-transfer characteristics [30]. Compared with the Isopar H case and the distilled water case, the result shows that RS distribution is significantly affected by different coolants. Because of the better cooling effects of using ethyl alcohol, the TRS on the machined surface decreases the ability of crack initiation, and thus increases the tensile strength even though it is also affected by surface quality [8]. The tensile strength has positive correlation with the related yield strength, namely $\sigma_{b}\propto \sigma_{0.2}$. It also indicates that ethyl alcohol can be used to obtain comparatively lager tensile strength in micromilling thin-walled Elgiloy.

As the material of the work piece has low thermal conductivity, the ability to take away heat of the coolants thus becomes more important. The evaporation process of coolants is an endothermic process and the evaporation rate of coolants has great impact on cooling performance. Considering the relative evaporation rate (n-BuAc = 100) of coolants, that of ethyl alcohol (202) is much larger than that of Isopar H (9) and distilled water (42). After the evaporation of ethyl alcohol droplets, heat is taken away and new ethyl alcohol droplets penetrate into the machining zone more easily. 

#### 3.3.4. The Effect of the Coolants on Breaking Elongation

**Figure 10:**
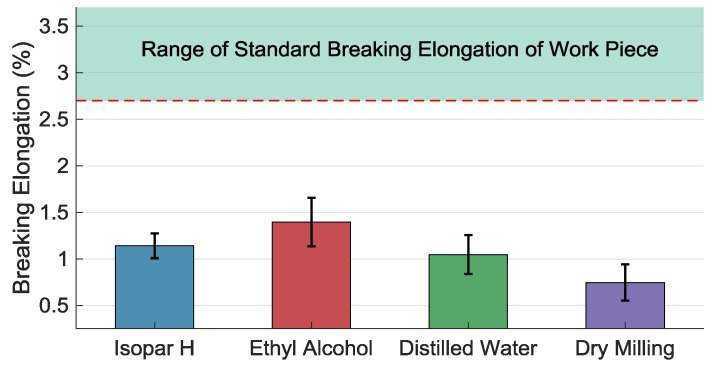
Breaking elongation of work piece micromilled under different coolants and methods.

Figure 10 shows the braking elongation *δ* of the machined work pieces under the four cases. All the *δ* are smaller than the minimum value of the standard breaking elongation, but *δ* under the ethyl alcohol case is the biggest one and that under the dry-machining case is the smallest one. Although the thickness of the machined work piece affects the breaking elongation, the machining process has great impact on the breaking elongation, as can be seen from Figure 10. It indicates that using ethyl alcohol obtains better surface integrity, such as a deformed layer and RS, and has the slightest impact on the breaking elongation. In this experiment, the breaking elongation *δ* under the Isopar H case is slightly larger than that under the distilled water case. It also indicates that the penetration and cooling effect of the coolants are more important to improve the machining process.

#### 3.3.5. Analysis on the Performance of the Coolants

**Figure 11:**
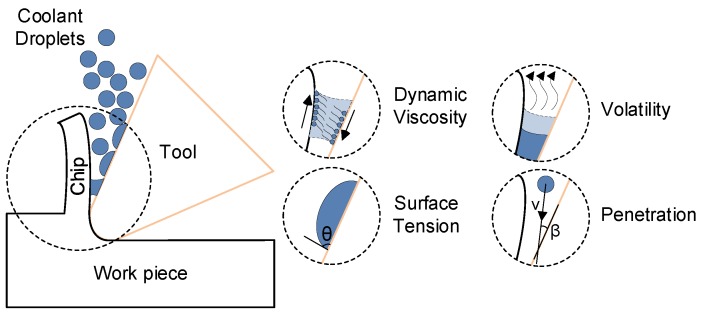
Schematic diagram of cooling and lubrication effect in a micromachining process.

In the case of spraying coolants into the interface between the chip and tool rake face (see Figure 11), the distance between the nozzle and the cutting zone, size, and moving direction of coolant droplets determine the penetration [18]. *v* denotes the velocity vector of coolant droplets and β denotes the angle between ***v*** and tool rake face. A smaller β has better penetration when the nozzle approaches tool rake face. However, it is complex in micromilling due to the complex geometry shape and rotational movement of the tool, so more than one nozzle should be adopted in the milling process. In addition, surface tension also affects coolant penetration. The contact angle θ always denotes wettability and smaller θ means better penetration [20].

In the lubrication and cooling process, dynamic viscosity denotes the frication coefficient between chip and tool surface. Coolant droplets with polar molecules adsorb the chip and tool surface, and friction in droplets happens when relative movement appears. The friction coefficient is then deduced. In this experiment, Isopar H belongs to chemisorption and the other coolants belong to physical absorption, so the adsorptivity of Isopar H is better. Isopar H possesses better performance on lubrication due to its high adsorptivity and low friction coefficient. In general, cooling effect is the main function to be the auxiliary method used in the macromachining process due to the large amount of heat generation during machining, and the lubrication effect is mainly used in the micromachining process. However, for a micromilling low thermal conductive work piece, especially in a very short reaction time, cooling effect is more important and that is also why a negative effect appears in machining Inconel 718 while using vegetable oil as lubrication [7]. The effect of convective heat transfer is larger than that of thermal radiation but smaller than the heat of evaporation in this micromilling process. As the saturated vapor pressure of ethyl alcohol is much larger than that of the other coolants, its relative volatility is the largest. The cooling effect of Isopar H is the worst in the experiment, but Isopar H has a good lubrication effect by which heat generation by friction is reduced, in terms of tensile experimental results. The relative volatility of ethyl alcohol is much larger than that of distilled water due to high good extreme pressure (EP) properties, even though the heat of evaporation (J/g) of distilled water is larger than that of ethyl alcohol. The generated heat is taken away by ethyl alcohol faster. 

## 4. Conclusions

In the micromilling process, auxiliary method MQCL has obvious impact on the machining results and mechanical properties of the component. Based on the experimental results, the conclusions that can be determined are as follows:

(1) In the dry-machining case, minor and major flaws on the surface are much more than those in MQCL cases. Even though MQCL has good performance on decreasing probability of BUE formation and improving surface quality, it cannot totally eliminate tool traces on the machined surface.
(2) Surface roughness in MQCL cases is decreased by a maximum of 10.4%, compared with that in dry-machining cases. The best surface roughness is generated in the Isopar H case, but the best surface accuracy appears in the ethyl alcohol case.
(3) The mechanical properties of the work piece after being micromilled are all smaller than the standard mechanical properties. The yield strength has positive correlation with the tensile strength of the machined work piece; however, the changing law of Young’s module is not obvious.
(4) Penetration of the coolants is important and is mainly affected by the physical characteristics of the coolant droplets. Combining the lubrication, penetration, and cooling effects of these coolants, in the selected coolants, ethyl alcohol is the most suitable one for micromilling thin-walled Elgiloy. Hence, to obtain good mechanical properties of a machined thin-walled work piece, cooling effect and penetration are more important, especially for machining materials with low thermal conductivity and high ductility.

## Figures and Tables

**Table 1:** Related information of the employed micromilling tool.

Dia. (μm)	Length of Cut (mm)	Neck Taper Angle (°)	Edge Radius (μm)	Material
≈150	0.2	9	≈2.5	WC

**Table 2:** Composition of Elgiloy (%).

Beryllium	Carbon	Chromium	Cobalt	Molybdenum	Iron	Manganese	Nickel
0.1 max	0.15 max	19–21	39–41	6–8	11.3–20.5	1.5–2.5	14–16

**Table 3:** Mechanical properties of Elgiloy.

Elasticity Module	Yield Strength	Tensile Strength	Breaking Elongation
(GPa)	(MPa)	(MPa)	(%)
189.6	1598–1667	1724–2413	2.7–3.7

**Table 4:** Micromilling parameters.

Spindle Speed (rpm)	Feed Rate (mm/s)	Milling Depth (μm × times)
40,000	1.67	10 × 5 + 5 × 2 + 1 × 5

**Table 5:** Micromilling method and the coolants used in MQCL.

	Work Piece Surface at Tool Total Cutting Length for			
Level	Isopar H (μm)	Ethyl Alcohol (μm)	Distilled Water (μm)	Dry (μm)
1	1320–1440	2760–2880	4200–4320	1320–1440
2	8520–8640	5640–5760	7080–7200	7080–7200
3	11,400–11,520	12,840–12,960	9960–10,080	12,840–12,960

**Table 6:** The tested mechanical properties of a standard component.

Young’s Module	Yield Strength	Tensile Strength	Breaking Elongation
(GPa)	(MPa)	(MPa)	(%)
189.59	1617.93	2065.21	3.39
